# Pilot study of new statistical models for prognostic factors in short term survival of oral cancer

**DOI:** 10.4314/ahs.v22i2.34

**Published:** 2022-06

**Authors:** Phillip Oluwatobi Awodutire, Oluwatosin Ruth Ilori, Chigozie Uwandu, Oladimeji Adeniyi Akadiri

**Affiliations:** 1 Department of Mathematics, University of Africa, Toru Orua; 2 Department of Community Medicine, LadokeAkintola University of Technology Teaching Hospital, Ogbomoso; 3 Department of Oral & Maxillofacial Surgery, University of Port Harcourt Teaching Hospital, Port Harcourt

**Keywords:** New statistical, short term survival, oral cancer

## Abstract

**Background:**

Survival times of oral cancer are poorly documented in Nigeria. This is partly due to poor documentation and limited investigations to elicit sufficient prognostic factors. In this study, we applied a new statistical model for survival times of oral cancer patients considering limited prognostic factors.

**Methods:**

A total of 29 cases of Oral cancer patients with stage I to IV invasive primary oral cancer treated at the University of Port Harcourt, Nigeria between 2008 and 2015 were used to generate prognostic models. Profiled prognostic factors include age, stage of tumor development, habitus, and treatment modalities. The baseline statistical distributions considered were Exponential, Weibull, Lognormal and Log-logistic distributions. The Chi-Square test was considered for the suitability of the model chosen. A comparison of the model performance was done using the Akaike Information Criterion (AIC).

**Results:**

Seventeen (58.6%) of the oral cancer patients were males and 12(41.4%) were females within the age range of 19 and 73 years. Sixteen (55.2%) of the patients were censored while 13(44.8%) were not censored. The estimated median survival time (MST) for the males was 29.50 months while that of the female was 7 months. Four parametric statistical models were tested and all identified tumor stage [cTNM stage (p= 0.000)] and treatment modality (p= 0.000) as more important predictors of survival. The models were then compared, using the Akaike Information Criterion (AIC) to determine the model best fit for the data. The model with the lowest AIC and so considered the best was the Weibull Statistical Model (WSM) with AIC= 100.76.

**Conclusions:**

This study suggests that the Weibull survival model is the best fit for estimating oral cancer survival times especially where only limited prognostic factors are available. Larger studies are required to validate the findings of this pilot.

## Introduction

A key measure of effective management of all types of cancer can be derived from survival analysis. However, large variations in survival data exist between cancer types and this can be adduced to numerous factors; ranging from tumor sites, histological type and biological behavior, treatment type, and effectiveness, and association with various genetic and molecular markers.^1^,^2^ While different survival rates have been described for the world's most common cancer types, a lot more is still required on a global scale, in terms of documentation and analysis of survival outcomes in oral cancers.^3^ Most cancer registries do not have as many extensive records on oral cancer as they do on other human cancer types.

In the United States of America, the SEER (Surveillance, Epidemiology and End Result program) database maintained by the National Cancer Institute (NCI)^3^ provides a resource for the American Cancer Society regarding survival statistics for different types of cancer. This database is however limited in terms of survival-related variables documented for oral cancers. For example, it does not relate oral cancer survival to some important prognostic factors such as American Joint Committee on Cancer(AJCC) Tumor Nose Metastasis(TNM) staging, treatment modalities, post excision tumor margin characteristics, significant biomarkers, etc. It only describes survival in relation to tumor spread i.e. localized, regional, or distant. The situation is worse in third-world countries where registration of oral cancers has not been properly mainstreamed into the national cancer registry.

In Nigeria, until 2009 when the Federal Ministry of Health (FMOH), Society of Oncology and Cancer Research of Nigeria (SOCRON), the Institute of Human Virology Nigeria (IHVN) coalesced to develop the National system of Cancer registries (NSCR)^4^, national cancer statistics had been abysmal. Since its inception, the NSCR has been able to revitalize defunct cancer registries and creating new ones across the states in the country. Unfortunately, however, most of these registries still pay little attention to oral cancer recording. For example, in the book^5^: “Cancer in Nigeria 2009 – 2013” published by the NSCR to document the prevalence of all cacer types in each state of the federation, very few states had any record on oral cancers. Rivers State in particular, with its cancer registry-based in the University of Port Harcourt Teaching Hospital, has a poor record of oral cancers managed in the hospital due to subconscious exteriorization of the oral and maxillofacial surgery department where these cases essentially present. The implication of all these is that there is a paucity of reports on the prevalence and survival of oral cancers from Nigeria in the global oncology literature. This study was therefore undertaken as a retrospective analysis to report the incidence of oral carcinomas at our clinic over 82 months (March 18, 2008 – December 15, 2015) and to determine a fitting statistical survival model best fit for the few prognostic factors retrieved from the patients' hospital records

## Materials and Methods

This is a retrospective study of cases of oral squamous cell carcinoma (OSCC) and adenocarcinomas presented for treatment at the Oral and Maxillofacial Surgery Clinic of the University of Port Harcourt Teaching Hospital Port Harcourt, Rivers State, Nigeria. Information obtained from the case files were patients' demographic features (age and gender), time of presentation (classified as early [<3 months of onset]) or late [>3 months of onset]), location of the tumor, morbidity status of the patient (based on the American Society of Anaesthesiologists' Classification [ASA I - V]), clinical tumor stage at presentation (cTNM stages I - IV), treatment modalities (Surgery, Radiotherapy, Chemotherapy or various combination therapy), follow-up period and survival outcome (dead, alive or lost to follow up). Tumour location (TL) was regrouped based on on-site contiguity because scanty cases per anatomic site were recorded. Hence, we had TL1 (palate/antrum Ca); TL2 (central mandibular Ca); TL3(gingiva, tongue/floor of the mouth); TL4 (Lip/cheek Ca) and TL5 (Parotid Ca). Due to uneven length and a high rate of loss to follow-up, the survival time was censored at 24 months from the day of presentation.

Statistical analysis was performed using SPSS version 21.0 and included a frequency analysis to profile patients according to the clinical factors examined and secondly, a survival analysis was done to determine the prognostic factors for 2 years short term survival. For the survival analysis, the parametric model approach was preferred to the Cox proportional hazard model and the Kaplan-Meier model due to the consistency and efficiency of the estimates of the parametric model. Furthermore, the parametric model approach is more appealing because of its ability to handle a small sample size of data. The parametric regression model (also known as Accelerated Failure time model AFT) is of the form

ln T = ν + d'β + s (1)

where s is said to follow a particular distribution, β's are the estimates of the covariates x, d is the coefficient of the covariates and ν is the intercept of the model. T is the time taken for the event to happen.

In this study, in investigating the relationship between the survival times of the patients and the prognostic factors, we employed four parametric models which are Exponential, Weibull, Log-normal, and Log-logistic survival models.

**Figure:**
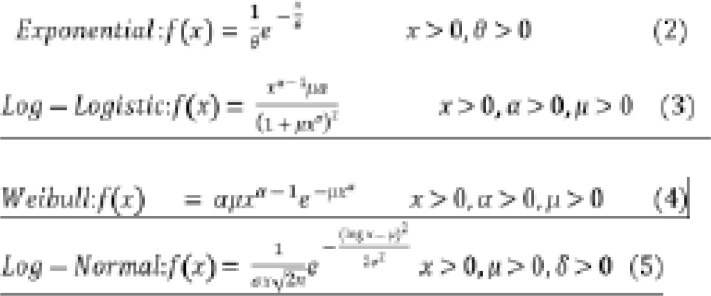


For model comparison, the Akaike Information Criterion (AIC) was used in which the model with the lowest AIC is considered the best 19. The AIC is given as equation (6).

AIC = 2k − 2 ln(L) (6)

Where L is the likelihood value of the model. Statistical significance was set at p-value<0.05.

## Results

**Figure 1:**
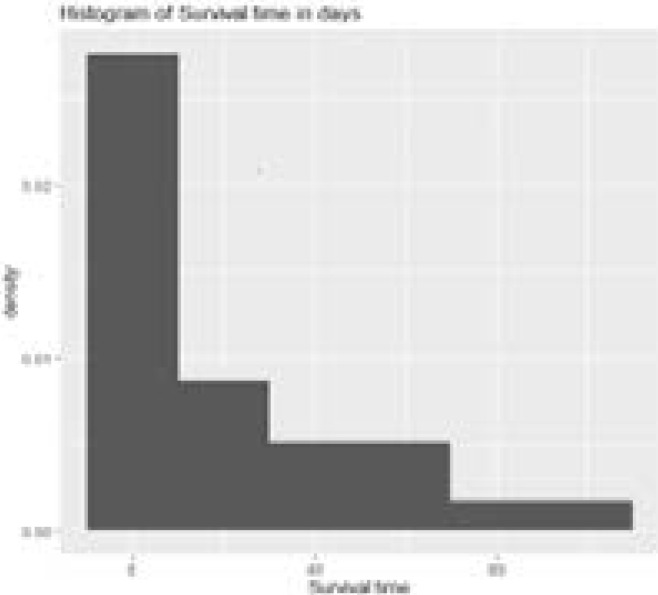
Survival Times of Oral Cancer Patients

**Figure 2:**
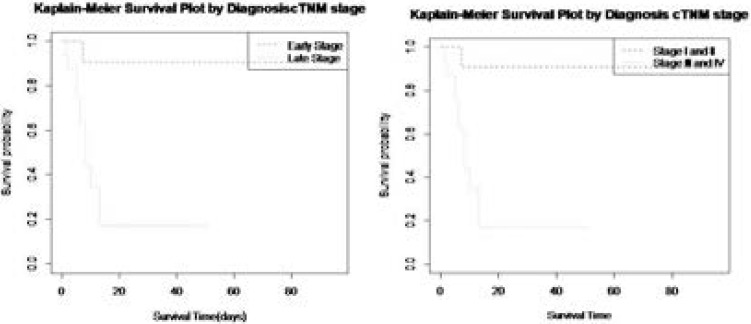
Kaplan-Meier curves of Oral Cancer Patients by cTNM stage

In this study, 29 cases of oral cancer patients were profiled including 17 (58.6%) males and 12(41.4%) females within the age range of 19 and 73 years. In terms of tumour location, we had TL1 (8; 27.6%), TL2 (10; 34.5%), TL3 (6; 20.7%), TL4 (3;10.3%), and TL5 (2;6.9%). There were 13(44.8%) early presentations and 16(55.2%) late presentations. Morbidity status as at the time of presentation were ASA I (9; 31.0%), ASA II (13; 44.8%) ASA III (3; 10.3%), ASA IV (4; 13.8%) while cTNM Stages were Stage I (4; 13.8%), Stage II, (7; 24.1%), Stage III, (8; 27.5%); Stage IV (10; 34.5%). Figure 1 reveals the distribution of the survival times of the patients. The shape of the plot implies that the data is rightly skewed, i.e as the survival times increase, the probability of surviving reduces. According to the survival curve in Figure 2, shows that the survival rate estimation by the Kaplan-Meier method in the different cTNM stages. During the first 8 months of follow-up, the survival probability is 100% for the Early cTNM stage and about 1 month for the Late cTNM stage. Table 1 depicts the Clinical staging (cTNM) and survival outcomes of the 29 patients analyzed with 18 (62%) of the patients presenting late at stage III and IV most of whom either died or were lost to follow-up. Table 2 shows that the majority of the patients either had surgery alone (13/29)or surgery combined with adjuvant radiotherapy (7/29), three patients had palliative non-oncological treatment. The goal of treatment was palliative in the majority of cases (19/29).

**Table 1:** Two years survival in relation to clinical TNM staging

cTNM stage	Outcome			Total No. of patients

	Alive N (%)	Dead N (%)	Lost to Follow-up N (%)	
Stage I	3 (30.0%)	-	1(8.3%)	4(13.8%)
Stage II	5 (50.0%)	-	2(16.6%)	7(24.1%)
Stage III	1(10.0%)	3(42.9%)	4(33.3%)	8(27.5%)
Stage IV	1(10.0%)	4 (57.1%)	5(41.7%)	10(34.5%)
**Total**	10 (100.0%)	7(100.0%)	12 (100.0%)	29(100.0%)

**Table 2:** Treatment modality, goals, and outcome

Treatment Modality	(N)	Goal of intervention	(N)	Outcome	(N)
Surgery alone	(13)	Palliative	(7)	Alive	(6)
		Cure	(6)	Dead	(2)
				LTF	(5)

Surgery-Radiotherapy	(7)	Cure	(4)	Alive	(2)
		Palliative	(3)	Dead	(3)
				LTF	(2)

Surgery- Chemotherapy	(2)	Palliative	(2)	LTF	(2)

Chemotherapy	(4)	Palliative	(4)	Alive	(1)
				Dead	(2)
				LTF	(1)

Non-oncological therapy	(3)	Palliative	(3)	Alive	(1)
				LTF	(2)

For the survival analysis, 16(55.2%) of the patients were censored while 13(44.8%) were not censored. The estimated median survival time (MST) for the males is 29.50 months while that of the female is 7 months. Using five survival models, namely; Exponential Survival Model (ESM), Weibull Survival Model (WSM), Log-normal Survival Model (L-nSM), Log-logistic Survival Model (L-lSM) and Cox Proportional Hazard Model(CPHM), the prognostic value of each of the profiled variables was determined (Table 3). Each of the models identified cTNM stage and treatment modalities as the two significant prognostic factors among the variables tested in this study. The four models were then compared, using the Akaike Information Criterion (AIC) to determine the model best fit for the data. The model with the lowest AIC and so considered the best was the WSM (AIC= 100.76). From the foregoing, therefore, treatment modality (p= 0.000) and cTNM stage (p= 0.000) were the two significant prognostic factors while the location of the tumor (p= 0.060) and morbidity status (p= 0.190) were insignificant in this study.

**Table 3:** Comparative results of four survival models

Model	Factors	Estimate	Standard Error	z	p
WSM	Age	0.002	0.01115	0.2	0.843
	Tumor Site	-0.343	0.180	-1.92	0.055
	cTNM stage	-2.851	0.720	3.96	0.00
	Patient Habitus	-0.378	0.295	-1.28	0.201
	Treatment Modality	0.473	0.112	4.22	0.00
	Loglik(model)= -44.5		AIC= 102.72		χ^2^=34.68(p=0.00)

ESM	Age				
	Tumor Site	-0.383	0.279	-1.37	0.1694
	cTNM stage	-3.594	1.124	-3.20	0.0014
	Patient Habitus	-0.405	0.422	-0.96	0.3370
	Treatment Modality	0.536	0.182	2.94	0.0033
	Loglik(model)= -46.2		AIC= 102.45		χ^2^=34.99(p=0.00)

L-lSM	Age	0.000762	0.012	0.06	0.95111
	Tumor Site	-0.273	0.217	-1.26	0.20919
	cTNM stage	-2.831	0.755	-3.75	0.00018
	Patient Habitus	-0.257	0.347	-0.74	0.45832
	Treatment Modality	0.422	0.137	3.07	0.00213
	Loglik(model)= -46.2		AIC= 106.33		χ^2^=28.17(p=0.00)

L-nSM	Age	0.0025	0.0138	0.18	0.8550
	Tumor Site	-0.2182	0.2389	-0.91	0.3612
	cTNM stage	-2.7251	0.6881	-3.96	0.0000
	Patient Habitus	-0.2582	0.3667	-0.70	0.4812
	Treatment Modality	0.4845	0.1589	3.05	0.0023
	Loglik(model)= -47		AIC= 107.91		χ^2^=25.32(p=0.00)

CPHM	Age	-0.033320	0.028372	-1.174	0.2402
	Tumor Site 0.001461	-	0.234899	-0.006	0.9950
	cTNM stage	3.615767	1.404731	2.574	0.0101
	Patient Habitus	0.476676	0.429575	1.110	0.2672
	Treatment Modality	-0.614786	0.247900	-2.480	0.0131
	Loglik(model)= -66.352		AIC= 142.704		LR
			=20.92(p=0.00)		

## Discussion

In domestic research, approximately 1.6% of cancer patients were diagnosed with malignant tumors in the oral and maxillofacial region; this was about 2,800 patients per year.^6^ Between the years 2000 and 2012, about 615,000 cases of oral cancer were reported, of which 300,000 were oral carcinomas.^7^ In fact, oral cancer is said to be the 16^th^ leading cause of cancer death worldwide with about 117,384 deaths per year.^8^ The actual figure is likely to be more, considering the poor reportage and grossly deficient archiving of oral cancer records in most national cancer registries, especially in the third world countries inclusive of Nigeria. In the present study, 29 cases of oral carcinomas are reported over almost 7 years. This gives an impression of approximately 4 cases per year which indeed is an underestimation of our experience. It is noteworthy that several cases were excluded from this study due to either scantiness of records or irretrievable record files. From the data analyzed, however, over 60% of the patient population presented late at clinical stage III and IV which accounted for the fatal outcomes. On the other hand, 80% of patients who survived, at least until 2 years after diagnosis, presented at either clinical stage I or II. The majority of the patients were lost to follow up thereby limiting the survival analysis to a short-term period of 2 years. This is the major bane of oral cancer survival epidemiology in our local environment.

A major question in the heart of a cancer patient and their relations borders on survival. Caregivers want to know their patient's chances of cure and survival, and for how long, if the cure is not guaranteed. The science behind the answers to these questions rests in the ability of the oncological team to assess valid prognoses based on the clinical features and factors presented by individual patients. Oral cancer prognosis, like other cancer types, depends on multiple variables. These include social, clinical, histological, molecular, and genetic variations associated with individual cases. In general, the predictor variables would include social habits such as tobacco and alcohol consumption, patient's age, gender, systemic health, regional lymph node status (i.e. presence/absence of metastasis), histologic grade of tumor (i.e. level of differentiation), tumor growth pattern (infiltrative or non-infiltrative), vascular /lymphatic invasion, perineural invasion (PI), extracapsular nodal extension, local recurrence, distant metastasis, interval to the detection of distant metastasis and quality of treatment.^9^–^11^ The quality of treatment includes especially; primary tumor resection and tumor margin clearance. Also, the quality of neck dissection and distant metastasis surgery such as pulmunectomy or laminectomy. Other prognostic treatment considerations are neoadjuvant/induction chemo-radiation, targeted therapy, or adjuvant therapies. In addition to these general factors, new molecular markers and genetic variations are being recognized as predictors of oral cancer prognosis.^10^ These include some MicroRNAs^11^,^12^, certain p53 mutations^13^, human papilloma viral oncogene^14^,^15^, and four new protein-signature (EGFR, HER2/neu, LAMC2 and RHOC)^16^ which have been associated with the risk of metastasis and poor prognosis. It has also been observed that expression of PD-L1 by oral squamous cell carcinoma is protective and associated with favorable survival outcomes in young females afflicted with the disease.^17^ PD-L1 is the ligand for the anti-apoptotic agent PD-1 which is now being used in targeted therapy against carcinomas. Some other host-related prognostic indices such as molecules, cells, and metabolites elaborated during the systemic inflammatory response to oral cancers are also emerging. These include C-reactive proteins, serum albumin, lymphocyte host reaction (LHR), and lymphocyte-monocyte ratio (LMR).^18^

Considering the plethora of prognostic indices now available, management of oral cancer patients now requires more robust evaluation to be able to assess the chances of cure and survival given the best possible treatment modalities. Unfortunately, in our local environment, there is still a huge limitation to the investigations that can be carried out for reasons ranging from economic to availability of expertise and /or facilities. The very basic investigations required for proper tumor staging such as nuclear medicine and fusion images of PET-CT and PET-MRI as well as advanced immuno-histochemical characterization of tumor histology are still difficult to come by. This often limits assessment to the pure clinical impression formed based on physical examination and basic radiological interrogations. In the present study, none of the patients had a PET scan therefore, the accuracy of tumor staging is very much in doubt; occult cervical nodal metastasis and distant metastasis may have been missed. In addition, only basic histological diagnoses based on hematoxylin and eosin staining were obtained in most cases thereby lacking further verification of specific tumor characteristics and behavior as well as prognostic host reaction factors that might influence the outcome and inform best treatment modalities. These enumerated limitations, coupled with deficient documentation characteristic of retrospective studies accounted for the very few prognostic factors examined in this study.

Cancer survival epidemiology is a very pertinent aspect of oncological research in which oral cancer research lags. This is especially so in the resource-poor countries of which Nigeria is an example. There is no single prior reference in oral carcinoma survival outcome studies in the entire Nigerian oncological literature. This study, therefore, forays into a virgin area in oral cancer research in Nigeria. It seeks to identify a statistical model that could fit the few variables available for assessing the prognosis of oral carcinoma in our practice.

Traditionally, Kaplan Meier survival estimate curves with log-rank test or Cox Hazard regression analysis are the major statistical models commonly used for survival analysis in the medical literature.^6^–^8^ Previous survival studies on oral carcinomas have also been based used these methods. In the present study, four alternative statistical survival models were tested and compared to determine the best fit among them. These survival models unlike the Kaplan Meier process in the multivariate equation to determine independent variables of significance. In this analysis, five variables were regressed by the models these are age tumor site, clinical stage (cTNM), patient habitus, and treatment modality. The four models identified clinical stage and treatment modality but when compared, the Weibull survival model was the best fit based on the Akaike information criterion. Being a single-center study, this finding may only have pertinent interpretation to the current practice in our facility and so may be useful for assessing patients' prognosis based on the management we currently offer. This is so because many other confounding factors were not entertained in the analysis either because the requisite investigations were not conducted or the information was not available/accessible. The high rate of loss to follow-up and consequent censoring of a considerable number of patients' data are additional limitations.

However, the findings signified, especially the clinical stage of the tumor is a well-established prognostic factor. On the other hand, the observation on treatment modality cannot be generalized as in most cases, the treatment was not standardized especially because a good number of our patients had to be referred out for adjuvant radiotherapy or chemo-radiotherapy in different centers, the majority of whom did not return. Hence the treatment modalities considered were mainly surgery and a few other cases involving adjuvant chemo and radiotherapy with a fairly reasonable post-treatment follow-up. Nevertheless, the Weibull survival model is recommended as a reliable alternative to the Kaplan Meier and log-rank test for determining survival outcomes in oral cancer. Because of the limitation of follow-up, only 2 years overall survival was estimated in this study while other survival measures such as disease-free survival (DFS) and disease-specific survival (DSS) were not estimated. However, the median survival time shows that the male gender has a better prognosis than females which is another fact well established in the oral cancer literature.

## Conclusion

In a pioneering effort, we have conducted a survival analysis of oral cancer patients in a single-center study. We have also demonstrated the effectiveness of the Weibull Survival Model as an alternative to the traditional Kaplan Meier method. A multicentre longitudinal study is required to further verify the variables that may be considered most useful for prognosticating the outcome of oral carcinoma in our local environment where extensive inquisition into advanced predictive factors based on nuclear imaging, immunohistochemistry, and molecular biomarkers is presently unattainable.
